# Estrogen stimulates female cancer progression by inducing myeloid-derived suppressive cells: investigations on pregnant and non-pregnant experimental models

**DOI:** 10.18632/oncotarget.26711

**Published:** 2019-03-08

**Authors:** Katsumi Kozasa, Seiji Mabuchi, Yuri Matsumoto, Hiromasa Kuroda, Eriko Yokoi, Naoko Komura, Mahiru Kawano, Ryoko Takahashi, Tomoyuki Sasano, Kotaro Shimura, Michiko Kodama, Kae Hashimoto, Kenjiro Sawada, Kazunori Nagasaka, Tadashi Kimura

**Affiliations:** ^1^ Department of Obstetrics and Gynecology, Osaka University Graduate School of Medicine, Osaka, Japan; ^2^ Department of Obstetrics and Gynecology, Teikyo University School of Medicine, Tokyo, Japan

**Keywords:** MDSC, estrogen, cervical cancer, brest cancer, pregnancy

## Abstract

**Objective:**

To investigate the clinical implications of 17β-estradiol (E2) in estrogen receptor α (ERα)-negative female cancer progression as well as the underlying biological mechanisms.

**Methods:**

Clinical data from 306 locally-advanced cervical cancer (stage IIB-IVA) patients were analyzed in order to investigate the relationships between age, serum E2 levels, and treatment outcomes. Clinical samples, ERα-negative cervical and breast cancer cell lines, and mouse xenograft models of cervical and breast cancers were employed in order to elucidate the mechanisms responsible for the E2- and pregnancy-mediated progression of cervical and breast cancers, with a focus on the role of myeloid-derived suppressor cells (MDSC).

**Results:**

Younger patients with elevated E2 levels showed significantly shorter progression-free survival (*P* = 0.040) and overall survival (*P* = 0.039). The exogenous E2 treatment stimulated the mobilization of MDSC from bone marrow and directly augmented their suppressive activities, leading to the progression of ERα-negative cervical and breast cancers. The co-administration of an anti-Gr-1 neutralizing antibody with E2 prevented the E2-mediated induction of MDSC, and attenuated E2-mediated tumor growth in cervical and breast cancer xenografts. Significantly increased MDSC numbers and enhanced tumor growth were observed during pregnancy in mice with cervical or breast cancer. Significantly increased MDSC numbers were also observed during pregnancy in cervical cancer patients.

**Conclusions:**

E2 facilitates the progression of ERα-negative cervical or breast cancer under non-pregnant and pregnant conditions by inducing MDSC. MDSC inhibition therapy may have therapeutic efficacy in premenopausal or pregnant female cancer patients.

## INTRODUCTION

Cervical cancer is the second most common female-specific cancer after breast cancer; an estimated 527,600 new cervical cancer cases and 265,700 deaths were reported worldwide in 2012 [1].

Estrogen is known to be involved in the development or progression of certain cancers: i.e., breast and endometrial cancers. Epidemiological studies reported that the majority of cervical cancer occurred in pre-menopausal women [2]. Moreover, the long-term use of oral contraceptives and high parity, both of which are indicative of long-term exposure to 17b-estradiol (E2), are risk factors for the development of cervical cancer [3, 4]. Although these findings indicate the involvement of E2 in cervical carcinogenesis, due to the lack of firm clinical evidence as well as mechanistic investigations, the role of E2 in the progression of cervical cancer has remained unclear. Consequently, cervical cancer has been clinically regarded as a hormone-independent tumor.

A recent Danish nationwide cohort study including 6135 cervical cancer patients showed that women diagnosed with cervical cancer during pregnancy (in which significant increases in E2 are observed) are at a higher risk of dying from cervical cancer than those who are not pregnant at the diagnosis of cervical cancer [5]. Similarly, 2 other groups also showed increased mortality for women diagnosed with cervical cancer during pregnancy [6, 7]. Moreover, recent studies using HPV transgenic mouse models provided preclinical evidence to show that E2 promotes the carcinogenesis and progression of cervical cancer [8, 9]. These findings strongly indicate that E2 plays important roles in the progression of cervical cancer.

Recent studies have suggested that the expression of estrogen receptor α (ERα) at the cell level markedly decreases during progression from normal epithelia to cervical cancer, whereas stromal ERα expression remains essentially unchanged [10]. The involvement of cancer-associated fibroblasts (CAFs), which express ERα, in the progression of cervical cancer was also demonstrated, indicating that E2 affects cervical cancer progression through ERα-expressing stromal cells.

ERα-expressing stromal cells exhibiting tumor-promoting activity are not only CAFs. Myeloid-derived suppressor cells (MDSC), a heterogeneous population of the myeloid lineage, are one of the stromal cells that express ERα [11]. MDSC have been shown to enhance tumor progression by suppressing tumor-specific T-cell responses, stimulating tumor angiogenesis, or promoting tumor cell metastasis [12]. Increased numbers of MDSC have been detected in the peripheral blood or tumor specimens of various cancer patients including those with cervical or breast cancer [13–15].

Recent studies reported that MDSC numbers were increased in pregnant women and contributed to the maintenance of pregnancy by inducing fetal-maternal tolerance [16, 17]. It is well known that E2 levels significantly increase during pregnancy [18]. Mauti *et al.* showed in a mouse study that breast tumors that developed during or shortly after pregnancy were highly metastatic [19], and that the suppressive activity of MDSC was responsible for the highly metastatic nature of breast cancer during pregnancy. Therefore, the presence of higher levels of MDSC during pregnancy may exert tumor-promoting effects in pregnant cancer patients. However, the mechanisms responsible for the increase in MDSC level during pregnancy in cancer patients have not yet been elucidated. Moreover, the role of MDSC in the progression of cervical cancer during pregnancy has yet to be investigated.

Therefore, we have conducted clinical and laboratory investigations using cell lines or mouse xenograft models of cervical/breast cancer, clinical tumor/blood samples, and patient clinical data. The specific aims of the present study are as follows: (a) to investigate the effects of an exogenous E2 treatment on the progression of ERα-negative female cancers, (b) to examine the impact of elevated endogenous E2 during pregnancy on the progression of ERα-negative female cancers, and (c) to elucidate the mechanisms by which E2 stimulates the progression of ERα-negative female cancers, with a focus on its effects on hematopoiesis and MDSC.

## RESULTS

### Prognostic significance of a younger age in cervical cancer patients

**Figure 1:**
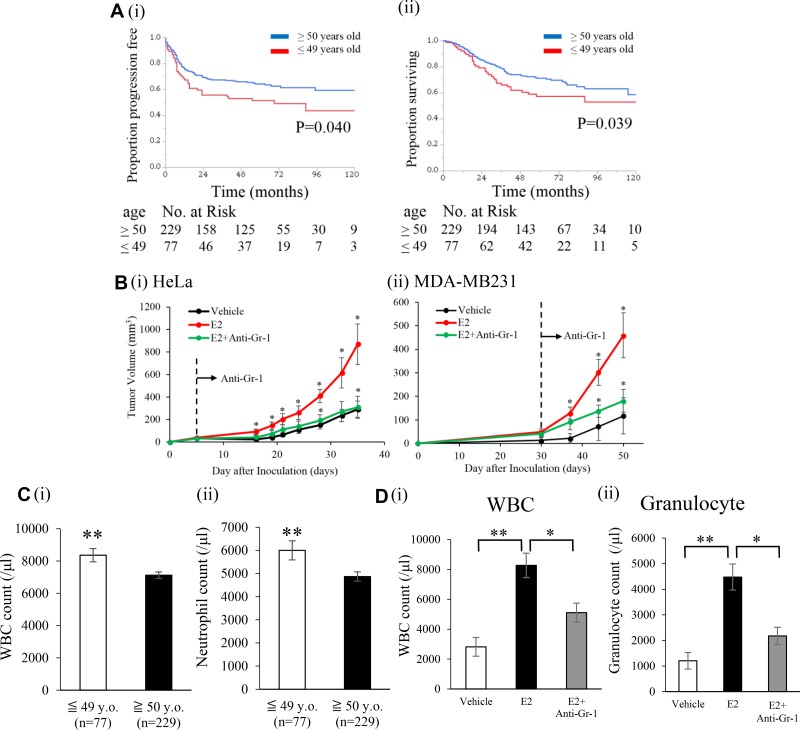
Effects of an exogenous E2 treatment on the progression of ERα-negative cervical/breast cancers (**A**) Kaplan–Meier estimates of survival according to age (*n* = 306). (i), Progression-free survival (PFS). PFS was significantly shorter in younger patients (≤ 49 years old, *n* = 77) than in older patients (≥ 50 years old, *n*=229). (ii), Overall survival (OS). OS was significantly shorter in younger patients (≤ 49 years old, *n* = 77) than in older patients (≥ 50 years old, *n* = 229). (**B**) Effects of E2 on the growth of cervical/breast cancers *in vivo*. Ovariectomized Balb/c nu/nu mice were inoculated with HeLa cells (ERα-negative cervical cancer cell line) or MDA-MB-231 cells (ERα-negative breast cancer cell line). Mice were treated with vehicle (0.1% ethanol) or E2 (10 μmol/L) in drinking water from the first day of the inoculation. An anti-Gr-1-neutralizing antibody or control IgG was administered intraperitoneally (150 μg/mouse) twice a week after the tumors reached a volume of approximately 30 mm^3^. (i) Growth curve of Hela-derived tumors, and (ii) growth curve of MDA-MB-231-derived tumors. Error bars, SE. ^*^P < 0.05 for vehicle vs E2 and E2 vs E2 with the anti-Gr-1-neutralizing antibody, Two-sided Student's *t*-test. (**C**) Impact of age on pretreatment blood cell counts in locally-advanced cervical cancer patients. (i) WBC counts and (ii) neutrophil counts assessed by an automated cell counter. Error bars, SE. ^**^P < 0.01, Two-sided Student's *t*-test. (**D**) Effects of the exogenous E2 treatment on blood cell counts in cervical cancer-bearing mice. (i) WBC counts and (ii) granulocyte counts. Error bars, SE. ^*^P < 0.05, ^**^P < 0.01, Two-sided Student's *t* test.

The clinicopathological characteristics of 306 locally-advanced cervical cancer patients (stage IIB-IVA) included in the present study are shown in Supplementary Table 1. Median age was 59 years old (range; 25-86). Since the median age of menopause in Japanese women is 50 years old, we divided patients into 2 groups: a younger age (<49 years old) and older age (> 50 years old). A younger age correlated with a high incidence of pelvic node metastasis (*P* = 0.0039) and non-SCC histology (*P* < 0.001) (Supplementary Table 1). As shown in Figure 1A, a younger age correlated with shorter progression-free survival (PFS) (*P* = 0.040) and overall survival (OS) (*P* = 0.039).

In order to elucidate the mechanisms responsible for the aggressive nature of cervical cancer in younger patients, using blood samples obtained from cervical cancer patients, we examined the relationship between age and serum 17β-estradiol (E2) concentrations. As shown in Supplementary Figure 1, as expected, E2 levels were significantly higher in younger patients than in older patients, indicating that E2 may play roles in cervical cancer progression.

### Effects of the exogenous E2 treatment on MDSC recruitment and the progression of ERα-negative cervical/breast cancers

Previous studies reported that the expression of ERα at the cell level markedly decreases during progression from normal epithelial cells to cervical cancer cells [10]. Thus, to investigate the effects of E2 on ERα-expressing stromal cells during cancer progression, we employed the ERα-negative cervical and breast cancer cells in the following experiments. As shown, Hela and MDA-MB-231 cells did not express ERα and did not show sensitivity to the E2 treatment, which is in clear contrast to ERα-expressing MCF7 (Supplementary Figure 2).

Using these ERα-negative cervical and breast cancer cell lines, we investigated the effects of the exogenous E2 treatment on tumor growth. As shown in Figure 1B, ovariectomized mice treated with E2 showed the significantly stimulated growth of cervical and breast cancers, which is consistent with the results obtained in cervical cancer patients. Moreover, importantly, this result indicates that E2-mediated progression of ERα-negative cancer is not specific for cervical cancer.

Since younger cervical cancer patients showed a significantly higher white blood cell (WBC) count, neutrophil count, and incidence of leukocytosis/neutrophilia than older patients (Figure 1C and Supplementary Figure 3), we hypothesized that the E2-mediated stimulation of granulopoiesis is involved in the progression of ERα-negative female cancers. In order to demonstrate this hypothesis, we investigated whether E2 stimulates granulopoiesis in mice. As shown, the exogenous E2 treatment resulted in significant increases in the WBC or granulocyte count in healthy and cancer-bearing mice (Figure 1D and Supplementary Figure 4A).

**Figure 2:**
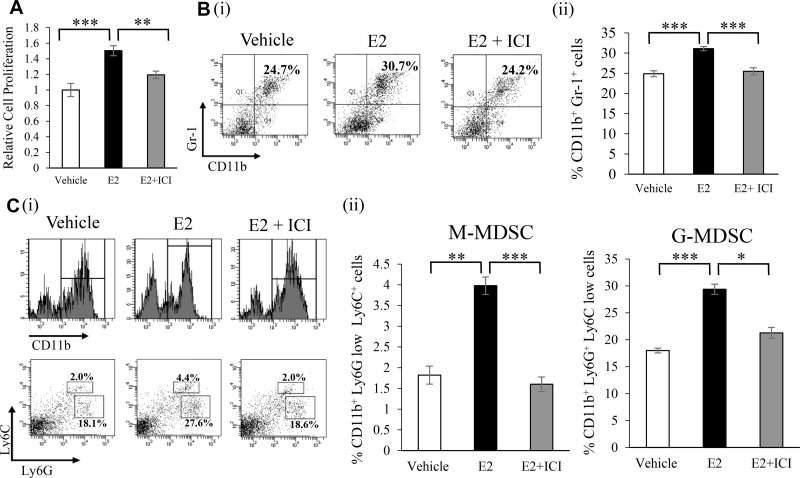
Effects of an exogenous E2 treatment on the induction of MDSC *in vitro* (**A**) Effects of E2 on the proliferation of BM cells. BM cells isolated from an ICR mouse were incubated with vehicle (0.1% ethanol) or 100 nM E2 in the presence or absence of 1 μM ICI 182,780 (estrogen receptor antagonist) for 72 hours. Cells were incubated with BrdU for an additional 24 hours, and cell proliferation was assessed by the incorporation of BrdU. Error bars, SE. ^**^P < 0.01, ^***^P < 0.001, Two-sided Student's *t*-test. (**B**) Effects of E2 on the differentiation of BM cells into MDSC. BM cells isolated from an ICR mouse were treated with vehicle (0.1% ethanol) or 100 nM E2 in the presence or absence of 1 μM ICI 182,780. The proportions of MDSC (CD11b+Gr-1+) were assessed by flow cytometry. (i) Representative dot plot. The percentages of MDSC were indicated. (ii) Frequencies of MDSC. Error bars, SE. ^***^P < 0.001, Two-sided Student's *t*-test. (**C**) Effects of E2 on MDSC subsets. *In vitro* BM cells isolated from an ICR mouse were treated with 100 nM E2 in the presence or absence of 1 μM ICI 182,780. The proportions of MDSC subpopulations were assessed by flow cytometry: CD11b+ cells were gated and then replotted for their Ly6G and Ly6C expression levels in order to assess the frequencies of the monocytic (CD11b^+^Ly6G^−^Ly6C^high^) and granulocytic (CD11b^+^Ly6G^+^Ly6C^low^) MDSC subsets. (i) Representative dot plots. The percentages of the MDSC subsets were indicated. (ii) Frequencies of MDSC subpopulations. Error bars, SE. ^*^P < 0.05, ^**^P < 0.01, ^***^P < 0.001, Two-sided Student's *t*-test.

**Figure 3:**
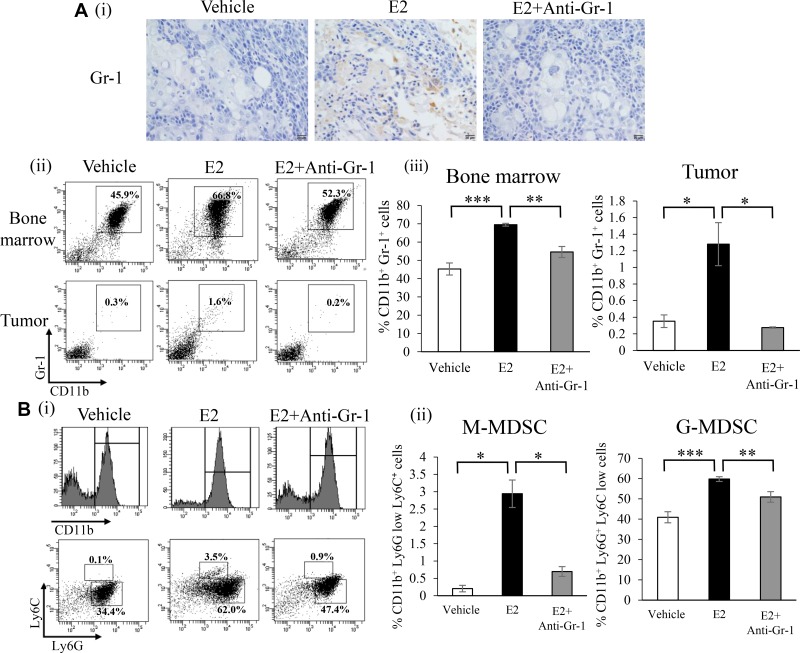
Effects of an exogenous E2 treatment on the induction of MDSC *in vivo* (**A**) Effects of E2 on the induction of MDSC in tumor-bearing mice. Immunostaining was performed using tumor samples obtained from vehicle-treated (*n* = 6), E2-treated (*n* = 6) and E2 plus anti-Gr-1 antibody-treated (*n* = 6) mice. Representative pictures were shown (magnification: ×400). (i) Representative immunohistochemical staining for Gr-1 in subcutaneous tumors (magnification: ×400). (ii) and (iii) Flow cytometric analysis of the proportion of MDSC in BM and tumors. Error bars, SE. ^*^P < 0.05, ^**^P < 0.01, ^***^P < 0.001, Two-sided Student's *t*-test. (**B**) Effects of E2 on MDSC subsets in tumor-bearing mice. (i) Representative dot plots. The percentages of MDSC subsets were indicated. (ii) The proportion of MDSC subsets in BM cells. Error bars, SE. ^*^P < 0.05, ^**^P < 0.01, ^***^P < 0.001, Two-sided Student's *t*-test.

We examined the effects of the E2 treatment on bone marrow (BM) cells. The proliferation of BM cells was significantly stimulated in response to the E2 treatment (Figure 2A). In order to elucidate the mechanisms by which the E2-mediated stimulation of granulopoiesis enhances the progression of female cancers, using mouse BM cells, we next performed an *in vitro* differentiation assay. As shown in Figure 2B, E2 stimulated the differentiation of BM cells into MDSC (CD11b^+^Gr-1^+^ cell population). When subpopulations of MDSC were examined, E2 increased monocytic MDSC (M-MDSC; CD11b^+^ Ly6G^−^ Ly6C^high^) and granulocytic MDSC (G-MDSC; CD11b^+^ Ly6G^+^ Ly6C^low^) MDSC *in vitro* (Figure 2C). Consistent with this result, ovariectomized healthy mice treated with E2 increased MDSC (Supplementary Figure 4B). Similar results were obtained in tumor-bearing mice (Figure 3A and Supplementary Figure 5). Moreover, as shown in Figure 3B, tumor-bearing mice treated with E2 had increased numbers of M-MDSC and G-MDSC, with G-MDSC being the dominant subset (Supplementary Figure 6). As shown in Supplementary Figure 7A, most MDSC obtained from tumor-bearing mice were recognized as granulocytes by the automated cell counter. Similarly, most human MDSC obtained from cervical cancer patients were recognized as neutrophils by the automated cell counter (Supplementary Figure 7B). When mice were treated with anti-Gr1 neutralizing antibody in combination with E2, E2-mediated increases in WBC, granulocytes in blood and MDSC (both M-MDSC and G-MDSC) numbers in the BM and tumors were almost completely abolished (Figure 1D, 3A, 3B), which resulted in marked decreases in the tumor burden (Figure 1B). Collectively, these results suggest that the exogenous E2 treatment stimulates the progression of ERα-negative female cancers by inducing MDSC.

### Mechanisms by which E2-induced MDSC stimulate the progression of female cancers

**Figure 4:**
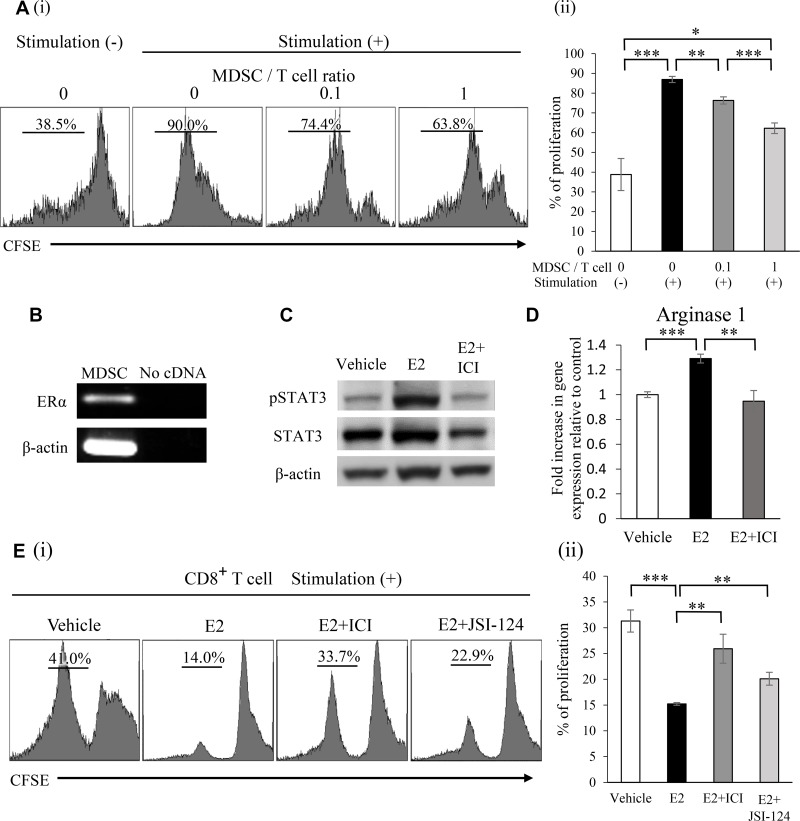
Mechanisms by which E2 stimulates the progression of female cancers (**A**) Ability of MDSC to suppress T cells. Mouse MDSC were isolated from the spleens of ME180-G-CSF-bearing Balb/c nu/nu mice. CD8+ T cells (2 × 10^5^ cells/well) were isolated from syngeneic mice. Mouse CD8+ T cells were labeled with CFSE and stimulated with the anti-CD3e antibody. Cells were co-cultured at the indicated ratio for 3 days and T-cell proliferation was evaluated by flow cytometry. Error bars, SE. ^*^P < 0.05, ^**^P < 0.01, ^***^P < 0.001, Two-sided Student's *t*-test. (**B**) The mRNA expression of ERα and β-actin in mouse MDSC. RNA isolated from mouse MDSC was assessed by RT-PCR. Each experiment was performed at least three times, and data from one representative experiment are shown. (**C**) Effects of E2 on STAT3 phosphorylation in MDSC. Mouse MDSC were treated with vehicle (0.1% ethanol) or 100 nM E2 in the presence or absence of 1 μM ICI 182,780 for 30 minutes. The phosphorylation of STAT3 in MDSC was assessed by Western blotting. Each experiment was performed at least three times, and data from one representative experiment are shown. (**D**) Effects of E2 on arginase I mRNA expression in MDSC. Mouse MDSC were incubated with vehicle (0.1% ethanol) or 100 nM E2 in the presence or absence of 1 μM ICI 182,780 for 60 minutes. Arginase I mRNA expression was then assessed by real-time RT-PCR. Error bars, SE. ^**^P < 0.01, ^***^P < 0.001, Two-sided Student's *t*-test. (**E**) Effects of E2 on the suppressive function of MDSC. CFSE-labeled CD8+ T cells were stimulated with the anti-CD3e antibody and co-cultured with MDSC at a 2:1 ratio in the presence or absence of E2, ICI 182,780, or JSI-124 (STAT3 inhibitor) for 3 days. T-cell proliferation was evaluated by flow cytometry. Error bars, SE. ^**^P < 0.01, ^***^P < 0.001, Two-sided Student's *t*-test.

We evaluated the immunosuppressive nature of MDSC used in our experimental models. As shown, mouse MDSC significantly inhibited T-cell proliferation (Figure 4A). Since the Janus kinase 2 (JAK2)-STAT3 pathway has been reported to play crucial roles in the activation of MDSC [20], we investigated the effects of E2 on STAT3 pathway activation in MDSC as well as on the proliferation of MDSC. As shown, ERα expression was observed in MDSC (Figure 4B). Consistent with previous findings [11, 16, 17], MDSC treated with E2 induced the significant activation of STAT3 via ERα (Figure 4C), leading to the increased production of Arginase I (Figure 4D). Moreover, MDSC treated with E2 significantly enhanced the suppressive effects of MDSC on CD8^+^ T cell proliferation (Figure 4E). These results indicate that E2 stimulates female cancer progression by inducing MDSC from BM and by enhancing their suppressive activity.

**Figure 5:**
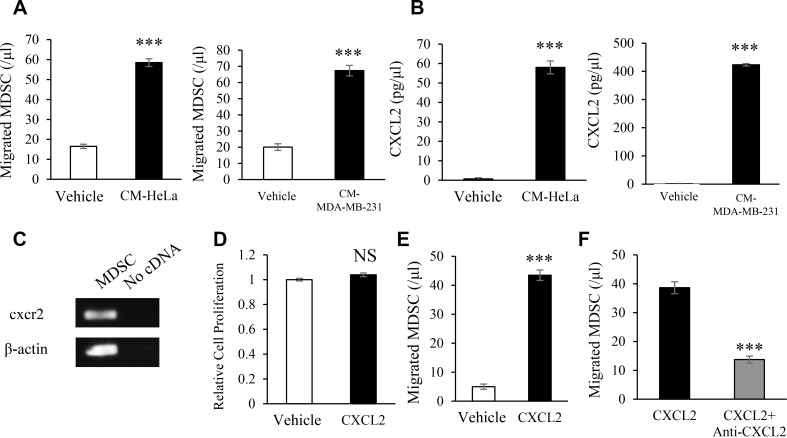
Mechanisms by which female cancers stimulate the induction of MDSC Effects of tumor-derived factors on the migration activity of MDSC assessed by a chemotaxis assay. (**A**) Mouse MDSC were seeded into the upper chamber of a Transwell system. Tumor-conditioned medium (CM) or unconditioned medium (control) was then added to the lower chamber. In order to obtain CM, supernatants were harvested from confluent cultures of HeLa or MDA-MB-231 cells. After a 2-hour inoculation, the number of migrated cells was counted. Error bars, SE. ^***^P < 0.001, Two-sided Student's *t*-test. (**B**) CXCL2 concentrations in CM measured by ELISA. Error bars, SE. ^***^P < 0.001, Two-sided Student's *t*-test. (**C**) Mouse cxcr2 mRNA expression in MDSC evaluated by RT-PCR. Each experiment was performed at least three times, and data from one representative experiment are shown. (**D**) Effects of CXCL2 on the proliferation of MDSC. Mouse MDSC were treated with or without mouse CXCL2 (100 ng/mL) for 48 hours. Cell proliferation was then assessed by the MTS assay. Error bars, SE. NS: not significant, Two-sided Student's *t*-test. (**E**) Chemotaxis of MDSC induced by mouse CXCL2. Error bars, SE. ^***^P < 0.001, Two-sided Student's *t*-test. (**F**) Effects of the anti-mouse CXCR2 antibody on the migration of MDSC. Mouse MDSC were seeded into the upper chambers of a Transwell system and incubated with the anti-mouse CXCR2 antibody (1 μg/mL) or IgG control antibody for 30 minutes. CXCL2 (100 ng/mL) was then added to the lower chamber. After a 2-hour inoculation, migrated cells were counted. Error bars, SE. ^***^P < 0.001, Two-sided Student's *t*-test.

We then investigated the mechanisms by which E2-induced MDSC migrate into female cancers. As shown (Figure 5A), MDSC treated with the conditioned medium of HeLa cells (CM-HeLa) and MDA-MB-231 (CM-MDA-MB-231) significantly stimulated the migratory activity of MDSC, indicating the involvement of Hela or MDA-MB-231 cell-derived soluble factors in the migration of MDSC. Based on previous findings showing that the interaction of the chemokine receptor CXCR2 expression on MDSC and cancer cell-derived chemokines play important roles in tumor progression [21], we confirmed the expression of chemokine (CXC motif) ligand 2 (CXCL2) in Hela and MDA-MB-231 cells (Figure 5B) as well as CXCR2 expression in MDSC (Figure 5C). Although MDSC treated with CXCL2 did not exhibit enhanced proliferation (Figure 5D), the CXCL2 treatment significantly stimulated the migration of MDSC (Figure 5E, 5F).

**Figure 6:**
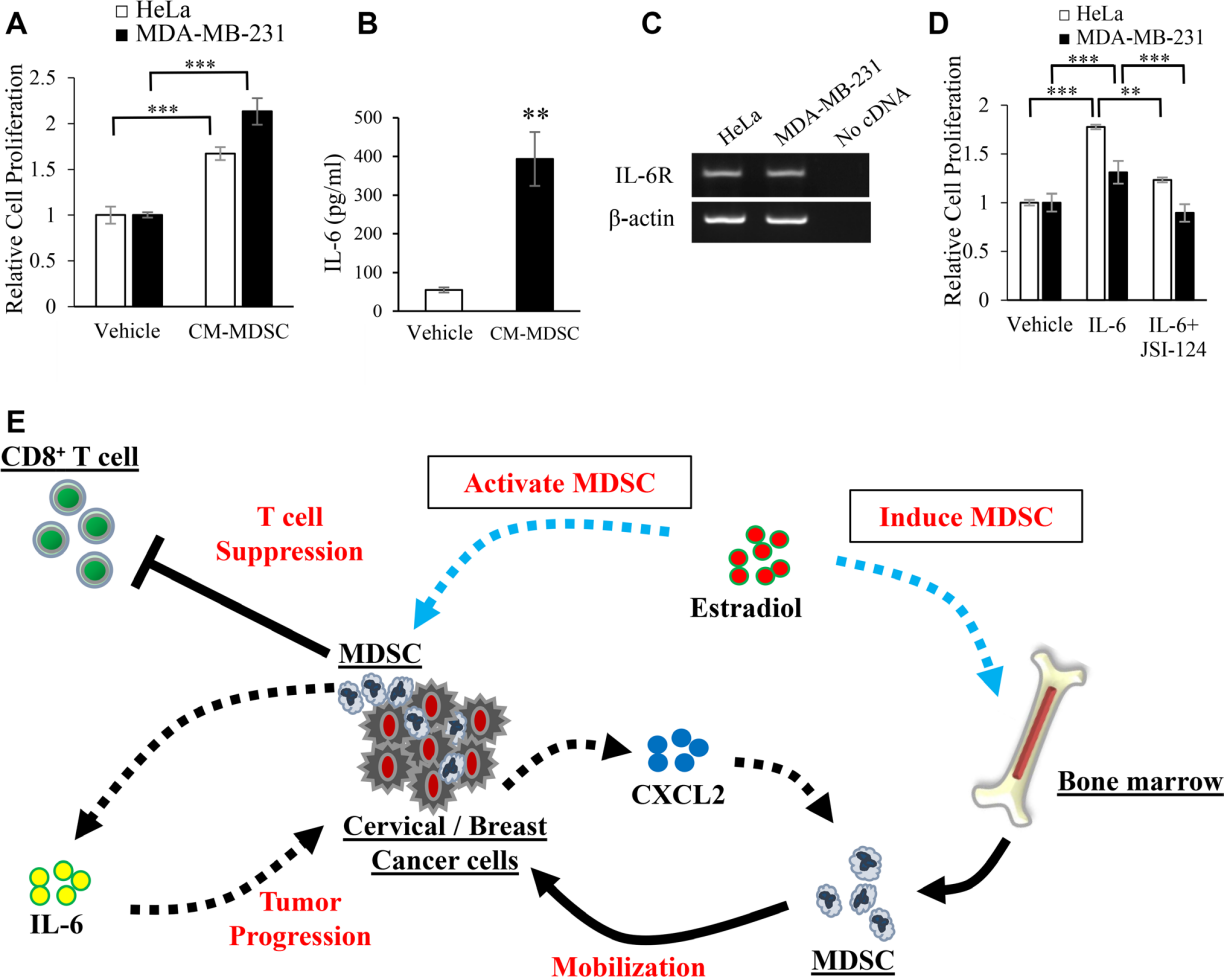
Mechanisms by which MDSC stimulate the progression of female cancers Effects of MDSC-derived factors on the proliferation of cervical and breast cancer cells. (**A**) HeLa and MDA-MB-231 cells were incubated with conditioned medium from MDSC (CM-MDSC) or unconditioned medium (control) for 48 hours. The proliferation of cancer cells was then assessed by the MTS assay. Error bars, SE. ^***^P < 0.001, Two-sided Student's *t*-test. (**B**) IL-6 concentrations in CM-MDSC assessed by ELISA. Error bars, SE. ^**^P < 0.01, Two-sided Student's *t*-test. (**C**) The mRNA expression of IL-6R in HeLa and MDA-MB-231 cells assessed by RT-PCR. Each experiment was performed at least three times, and data from one representative experiment are shown. (**D**) Effects of IL-6 on the proliferation of HeLa and MDA-MB-231 cells. HeLa and MDA-MB-231 cells were incubated in the presence or absence of IL-6 (100 ng/mL) or JSI-124 (100 nM) for 48 hours, and proliferation was assessed by the MTS assay. Error bars, SE. ^**^P < 0.01, ^***^P < 0.001, Two-sided Student's *t*-test. (**E**) Proposed mechanism by which E2 stimulates the progression of cervical/breast cancers.

We then examined the effects of MDSC on the proliferation of tumor cells. As shown in Figure 6A, HeLa and MDA-MB-231 cell proliferation was stimulated when incubated with the conditioned medium of MDSC (CM-MDSC). Since CM-MDSC contained significant concentrations of IL-6 (Figure 6B) and HeLa and MDA-MB-231 cells expressed IL-6 receptor (IL-6R) (Figure 6C), we investigated the effects of IL-6 on the proliferation of Hela and MDA-MB-231 cells. As shown in Figure 6D, the IL-6 treatment significantly enhanced the proliferation of HeLa and MDA-MB-231 cells. These results indicated that the increase in cancer cell proliferation induced by CM-MDSC was mediated, at least in part, by MDSC-derived IL-6. Collectively, these results from human and mouse studies indicate that E2 drives the mobilization of MDSC from BM, directly augments their activity via ERα, and facilitates the progression of ERα-negative cervical or breast cancer (Figure 6E).

### Effects of increased endogenous E2 levels during pregnancy on MDSC recruitment and the progression of ER-negative cervical/breast cancers

**Figure 7:**
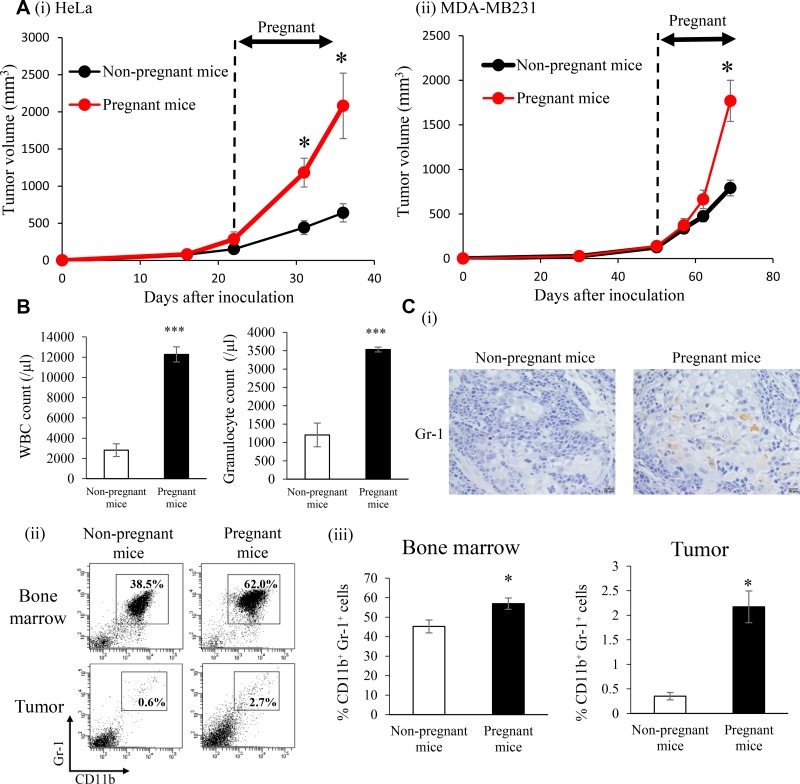
Effects of increased endogenous E2 levels during pregnancy on the recruitment of MDSC and progression of ERα-negative cervical/breast cancers *in vivo* SCID mice were subcutaneously inoculated with HeLa or MDA-MB-231 cells. When tumors reached a volume of approximately 100 mm^3^, half of the mice were impregnated. On day 18 of gestation, mice were killed and BM cells, tumor cells, and PBMC were collected for analyses. (**A**) Tumor growth curves: (i) Hela cells, (ii) MDA-MB-231 cells. Error bars, SE. ^*^P < 0.05, Two-sided Student's *t*-test. (**B**) WBC/granulocyte counts in pregnant and non-pregnant HeLa-derived tumor-bearing mice. Error bars, SE. ^***^P < 0.001, Two-sided Student's *t*-test. (**C**) (i) Gr-1 expression in HeLa-derived tumors in pregnant (*n* = 6) and non-pregnant (*n* = 6) mice. Representative pictures were shown (magnification: ×400). (ii)(iii) MDSC (CD11b+Gr-1+ cells) in pregnant and non-pregnant HeLa-derived tumor-bearing mice. Error bars, SE. ^*^P < 0.05, Two-sided Student's *t*-test.

**Figure 8:**
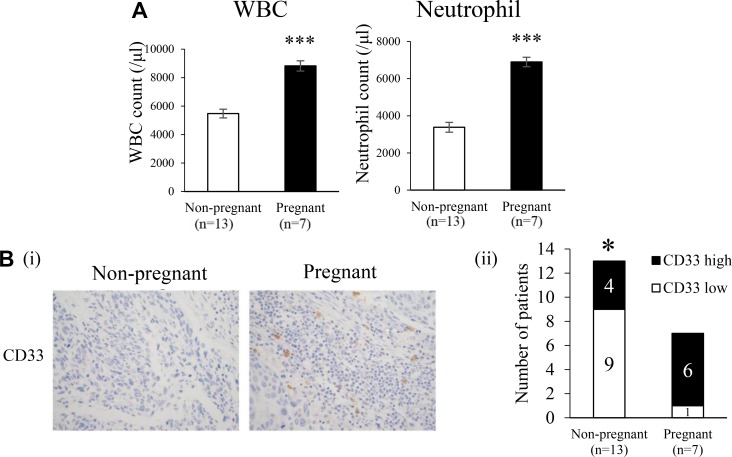
Effects of pregnancy on myelopoiesis and induction of MDSC in cervical cancer patients (**A**) Pretreatment WBC and neutrophil counts in pregnant and non-pregnant cervical cancer patients. Error bars, SE. ^***^P < 0.001, Two-sided Student's *t*-test. (**B**) CD33 expression in human cervical cancer. Cervical tumors were obtained from pregnant cervical cancer patients (*N* = 7) and age-matched non-pregnant cervical cancer patients (*N* = 13). Tumors were fixed, stained with the anti-CD33 antibody, and the number of tumor-infiltrating CD33^+^ cells was counted. Patients were classified into 2 groups: CD33^+^ high and CD33^+^ low, based on the median number of tumor-infiltrating CD33^+^ cells (i) Representative immunohistochemical staining for CD33 (magnification: ×400). (ii) The proportions of CD33+ high and CD33^+^ low patients. ^*^P < 0.05, χ^2^ test.

It is well known that E2 levels significantly increase during pregnancy [18]. Thus, we investigated the effects of increased endogenous E2 levels during pregnancy on the progression of ERα-negative cervical and breast cancers. As shown, serum E2 levels significantly increased during pregnancy in non-cancer-bearing mice (Supplementary Figure 8A). Consistent with the results shown in Supplementary Figure 4, WBC, granulocytes, and MDSC also increased in non-cancer-bearing pregnant mice (Supplementary Figure 8B, 8C). Importantly, as shown in Figure 7A, tumor growth in cancer-bearing mice was significantly enhanced during pregnancy. The increases observed in WBC and granulocytes were also confirmed in cancer-bearing mice during pregnancy (Figure 7B). Furthermore, higher numbers of MDSC in BM and subcutaneous tumors were found in pregnant cancer-bearing mice than in non-pregnant cancer-bearing mice (Figure 7C and Supplementary Figure 9). In order to confirm whether the results obtained in mice are representative of the clinical status of cervical cancer patients, we performed immunohistochemical analyses using patient-derived tumor specimens obtained before the initiation of treatment. As shown in Figure 8A, higher WBC and neutrophil counts were observed in pregnant cervical cancer patients than in non-pregnant cervical cancer patients. Moreover, higher numbers of tumor-infiltrating MDSC were observed in pregnancy-complicated cervical cancer than in non-pregnant cervical cancer (Figure 8B).

## DISCUSSION

We herein demonstrated that an exogenous E2 treatment and increase in endogenous E2 levels during pregnancy drove the mobilization of MDSC from the BM, directly augmented their immunosuppressive activity via ERα, and facilitated the progression of ERα-negative cervical/breast cancers.

Our results are consistent, at least in part, with a recent study suggesting that the estrogen-induced up-regulation of myelopoiesis results in the mobilization of MDSC from the BM and drives the progression of ovarian cancer [11]. The present results also appear to be consistent with previous findings showing the importance of stromal estrogen signaling in cervical cancer [10].

Our results may have important clinical implications. Our results provide insights into the gender-dependent mechanisms of cancer progression. Up-regulated myelopoiesis induced by estrogen results in the induction of MDSC, leading to the progression of two major female cancers, breast and cervical cancers. In order to directly demonstrate the effects of estrogen on MDSC-mediated tumor growth, the present study employed only ERα-negative female cancer cell lines. However, the estrogen-MDSC axis demonstrated in the present study should also work in ER-positive female cancers and enhance tumor progression in concert with the well-known estrogen-tumor ERα axis. Our results also raise concerns for the use of estrogen replacement therapy in female cancer patients. Estrogen replacement therapy is contraindicated in patients with breast cancer. Regarding cervical cancer, although women with adenocarcinoma given estrogen replacement therapy were found to be at a greater risk of recurrence in a case-control study [22], since there is currently no robust randomized-controlled evidence to suggest higher recurrence in cervical cancer patients receiving estrogen replacement therapy after initial cancer treatments, hormone replacement therapy has not been contraindicated in cervical cancer patients with menopausal symptoms. Our mechanistic investigations as well as previous clinical studies suggest that prolonged exposure to a high level of estrogen paradoxically enhances tumor growth. Thus, the cautious use of estrogen is recommended in female cancer patients, particularly those who are at high risk of developing recurrent/persistent disease after initial treatments. Further clinical trials investigating the oncological safety of estrogen replacement therapy in cervical cancer patients are desirable. The results of the present study may provide an explanation for obesity being a poor prognostic factor in cervical/breast cancer patients because obesity is associated with increased estrogen production from adipocytes [23, 24], or a high-fat diet promotes tumor progression by inducing MDSC [25]. Collectively, these findings indicate that increases in estrogen levels in women caused by estrogen replacement therapy, pregnancy, or obesity are associated with changes in the immune system, which may ultimately stimulate the progression of female cancers. Our results also provide a novel insight into immunosuppression during pregnancy. Recent studies on humans and mice revealed increased numbers of MDSC in the cord blood, peripheral blood, and uterus of healthy pregnant women or mice [26–28], indicating that MDSC represent a major player in fetal-maternal tolerance. In contrast to this favorable effect on pregnant women (i.e., preventing abortion), our results indicate that estrogen-induced MDSC paradoxically facilitates the progression of female cancers during pregnancy. This result may be consistent with and provide a possible explanation for the case series of cervical or breast cancers exhibiting rapid tumor progression during pregnancy [29, 30]. The present results also provide a rationale for new female cancer treatment strategies: targeting estrogen, MDSC, or downstream effectors of MDSC. To date, no specific inhibitor of human MDSC has been developed. However, anti-estrogen or chemokine inhibitors are available for human use. Thus, in non-pregnant women, the use of anti-estrogens, such as tamoxifen, or downstream effectors of MDSC, including the CXCL2/CXCR2 axis, may be practical and promising. The clinical activity of estrogen or MDSC-targeting treatments alone or in combination with conventional treatments, such as chemotherapy or radiotherapy, need to be investigated in future studies. In pregnant women, anti-estrogen therapy, such as the use of tamoxifen, is contraindicated because studies on mice or post-marketing data have demonstrated fetal risks, including a low birth weight, spontaneous abortions, birth defects, feto-toxicity, and still births [31]. Based on the role of MDSC in fetal-maternal tolerance in pregnant women, reasonable treatment strategies may be downstream effectors of MDSC (i.e., targeting the CXCL2/CXCR2 axis). The potential risks of this treatment need to be investigated in the future.

The limitations of the present study need to be addressed. Clinical investigations in the present study were retrospective and conducted at a single institution. Furthermore, due to the rarity of pregnancy-complicated cervical cancer cases, we were unable to investigate the impact of pregnancy on the prognosis of cervical cancer patients. Thus, a collaborative multi-institutional investigation, particularly in a prospective setting, is desirable. Another limitation is that we employed an anti-Gr-1-neutralizing antibody to deplete MDSC in the present study. However, we cannot rule out the possibility that the anti-Gr-1-neutralizing antibody also affects other cells, such as neutrophils. Moreover, although the present study focused on estrogen-induced MDSC, we cannot exclude the possibility that other stromal cells, such as tumor-associated neutrophils (TAN) or tumor-associated macrophages (TAM), also play roles in the progression of cervical cancer in an estrogen-dependent manner. Accordingly, the mechanisms by which estrogen stimulate cervical cancer progression need to be investigated in more detail.

In conclusion, we herein demonstrated that an exogenous E2 treatment and increase in endogenous E2 levels during pregnancy drive the mobilization of MDSC from BM and facilitate the progression of ERα-negative cervical or breast cancers. Our results provide a scientific rationale for future clinical trials on MDSC-inhibition therapy or anti-estrogen therapy in patients with ERα-negative female cancers.

## MATERIALS AND METHODS

### Patients and clinical samples

A list of patients who were diagnosed with FIGO stages IIB-IVA cervical cancer and treated at Osaka University Hospital between 1993 and 2011 was generated from our institutional tumor registry, and their clinical data were retrospectively analyzed. Tumor/blood samples were collected from cervical cancer patients. Appropriate informed consent was obtained from each patient. Follow-up examinations after the initial treatment were performed by gynecological and/or radiation oncologists at regular intervals at an outpatient clinic, as reported previously [32].

### Cell lines

Human cervical cancer cell lines (HeLa and ME180) and human breast cancer cell lines (MDA-MB-231 and MCF7) were purchased from the American Type Culture Collection. Cells were passaged soon after they were received from the cell bank, before being divided and stored in liquid nitrogen vessels. Each experiment was performed using thawed cells without further authentication. Cells were routinely screened for mycoplasma species (EZ-PCR Mycoplasma Test Kit; Biological Industries, Cromwell, CT, USA). Each experiment was carried out using thawed cells without further authentication. Cells were maintained in Dulbecco's Modified Eagle's Medium (DMEM) supplemented with 10% fetal bovine serum (FBS) in an atmosphere containing 5% CO_2_ at 37°C. However, when examining the effects of E2 on cell proliferation, cells were cultured in phenol red-free DMEM supplemented with 10% FBS in an atmosphere containing 5% CO_2_ at 37°C.

### Reagents/antibodies

The following labeled monoclonal antibodies were used in staining experiments: anti-human antibodies: V450-conjugated anti-CD33 (eBiosciences, San Diego, CA, USA) and allophycocyanin-conjugated anti-human leukocyte antigen (HLA)-DR (Biolegend, San Diego, CA, USA); anti-human/mouse antibodies: fluorescein isothiocyanate (FITC)-conjugated anti-CD11b (Tonbo Biosciences, San Diego, CA, USA); anti-mouse antibodies: phycoerythrin (PE)-conjugated anti-Gr-1 (R&D systems, Minneapolis, MN, USA), allophycocyanin (APC)-conjugated anti-Ly6G (Tonbo Biosciences, San Diego, CA, USA), and PE-conjugated anti-Ly6C (Tonbo Biosciences, San Diego, CA, USA). A neutralizing antibody against Gr-1 (RB6-8C5) was purchased from BioXCell (West Lebanon, NH, USA). E2 was purchased from Sigma Chemical Co. (St Louis, MO, USA). An estrogen receptor (ER) antagonist, ICI 182,780, was purchased from Tocris Bioscience (Bristol, UK). An anti-CD3e antibody was purchased from Tonbo Biosciences (San Diego, CA, USA). Antibodies against STAT3, phospho-STAT3 (Tyr705), and β-actin (Cell Signaling Technology, Beverly, MA, USA) were used in the Western blotting analysis. The STAT3 inhibitor, JSI-124 (cucurbitacin I), was purchased from Calbiochem (San Diego, CA, USA). Recombinant mouse CXCL2 was purchased from Wako (Osaka, Japan). An anti-mouse CXCR2 antibody was purchased from R&D systems (Minneapolis, MN, USA). Recombinant human interleukin (IL)-6 was purchased from Pepro Tech Inc. (Rocky Hill, NJ, USA). G-418 was purchased from Life Technologies (Grand Island, NY, USA).

### Enzyme-linked immunosorbent assay (ELISA)

Serum E2 concentrations were analyzed using an enzyme-linked immunosorbent assay (ELISA) kit (Cayman Chemical, Ann Arbor, Michigan, USA), according to the manufacturer's protocol. Human CXCL2 and mouse interleukin (IL)-6 concentrations in HeLa-, MDA-MB-231-, or MDSC- conditioned medium were analyzed by an ELISA kit (Abcam, Cambridge, UK, and eBioscience, San Diego, CA, USA) according to the manufacturer's protocol.

### Reverse transcriptase polymerase chain reaction (RT-PCR)

RNA was extracted from cells using TRIzol (Life Technologies, Grand Island, NY, USA). The resultant total RNA (1 μg) was used to synthesize cDNA with ReverTraAce qPCR RT Master Mix (Toyobo, Osaka, Japan). PCR was conducted using TaqMan PCR master mix (Qiagen, Valencia, CA, USA) and specific primers. Amplification was performed using a Takara PCR personal-type thermal cycler (Takara, Shiga, Japan). PCR primers were purchased from Life Technologies (Grand Island, NY, USA). The sequences of the primers used were as follows: β-actin: forward primer, 5′-CGTGACATTAAGGAGAAG CTGTG-3′ and reverse primer, 5′-GCTCAGGAGGAG CAATGATCTTGA-3′; human ERα : forward primer, 5′-GA ATCTGCCAAGGAGACTCG-3′ and reverse primer, 5′-CA GCATCCAACAAGGCACT-3′; mouse ERα : forward primer, 5′-GCCCTCCCGCCTTCTACA-3′ and reverse primer, 5′-CCCTCCTCGGCGGTCTTT-3′; mouse CXCR2 : forward primer, 5′-TGTTCTTTGCCCTGACCTTGC-3′ and reverse primer, 5′-ACGCAGTACGACCCTCA AACG-3′; human IL-6 receptor (IL-6R) : forward primer, 5′-CATTGCCATTGTTCTGAGGTTC-3′ and reverse primer, 5′-GTGCCACCCAGCCAGCTATC-3′.

### Cell proliferation assay

The MTS assay was used to analyze (1) the effects of E2 on the proliferation of HeLa, MDA-MB-231, and MCF7, and (2) the effects of the conditioned medium of MDSC (CM-MDSC) and IL-6 on the proliferation of HeLa and MDA-MB-231. Cell lines were plated on 96-well plates and exposed to E2, CM-MDSC, or IL-6. After a 48-hour incubation, the number of cells was measured by assessing the absorbance of the dissolved formazan product at 490 nm, as described by the manufacturer (Promega, Madison, WI, USA).

The BrdU assay was used to analyze the effects of E2 on the differentiation of bone marrow (BM) cells, as described previously [15, 33]. BM cells were plated on 96-well plates and exposed to vehicle (0.1% ethanol) or E2 (100 nM) with or without ICI 182,780 (1 μM). After a 48-hour incubation, cell proliferation was assessed using a cell proliferation ELISA BrdU kit (Roche Applied Science, Penzberg, Germany). Cell viability is expressed as follows: absorbance of the experimental group/absorbance of the control group.

### *In vivo* tumor studies

All procedures involving mice and their care were approved by the Institutional Animal Care and Usage Committee of Osaka University in accordance with institutional and National Institutes of Health guidelines. The first set of experiments was performed in order to examine the effects of E2 on the induction of MDSC and tumor growth. Female Balb/c nu/nu mice aged 5-6 weeks were surgically ovariectomized after anesthesia, and were allowed to recover for 3 weeks. They were then inoculated with 1.5 × 10^7^ HeLa (ERα-negative cervical cancer cell line) or MDA-MB-231 (ERα-negative breast cancer cell line) in 150 μL of Matrigel. They were treated with vehicle (0.1% ethanol) or E2 (10 μmol/L) in drinking water from the first day of the inoculation. The anti-Gr-1-neutralizing antibody or control IgG treatment was initiated and administered intraperitoneally at 150 mg/mouse twice a week after tumors reached a volume of approximately 30 mm^3^ and tumor growth was observed. The second set of experiments was performed in order to examine the effects of pregnancy on the induction of MDSC and tumor growth. Female SCID mice aged 5-6 weeks were subcutaneously inoculated with 1.5 × 10^7^ HeLa or MDA-MB-231 cells in 150 μL of Matrigel. They were impregnated after tumors reached a volume of approximately 100 mm^3^ and tumor growth was observed. At the end of both experiments, mice were killed by carbon dioxide asphyxiation, and samples were collected for analyses. Pregnant mice were killed on day 18 of gestation. Tumor growth was assessed in three dimensions using calipers, and tumor volume was calculated using the formula V = π/6 × L ×W × D, where V is volume, L is length, W is width, and D is depth.

### Blood cell count

Mouse WBC, granulocytes, and MDSC were counted with a VetScan HM2 automatic cell counter (Abaxis, Union City, CA, USA). Human WBC, granulocytes, and MDSC were counted using Sysmex EX-5000 (Sysmex Corporation, Kobe, Japan).

### Flow cytometry

Single cell suspensions were prepared from blood/tumor samples obtained from cervical cancer patients or tumor-bearing mice. Red blood cells were removed using ammonium chloride lysis buffer. Cells were then filtered through 40-μm nylon strainers, incubated with antibodies, and analyzed by flow cytometry. Flow cytometric data were acquired on a FACSCanto II or FACSAria II flow cytometer and analyzed using FACS Diva software (BD Biosciences, San Jose, CA, USA). Cells that had been incubated with irrelevant isotype-matched antibodies and unstained cells served as controls.

### Isolation of BM cells

BM from mice was flushed from the medullary cavities of femurs using a 26G needle. Red blood cells were removed using ammonium chloride lysis buffer. Cells were dispersed by gentle aspiration with a Pasteur pipet, centrifuged at 150 × *g* for 5 minutes, and resuspended in each medium or phosphate-buffered saline (PBS) containing 4% FBS.

### Immunohistochemistry

Tumor samples obtained from cervical cancer patients or tumor-bearing mice were fixed in 10% neutral buffered formalin, embedded in paraffin, sectioned, and processed for immunohistochemical staining. Mouse tumor tissues were stained with an anti-Gr1 antibody (R&D systems, Minneapolis, MN, USA) and human tumor tissues were stained with an anti-CD33 antibody (Leica Biosystems Inc., Buffalo Grove, IL, USA), as reported previously [34]. Optical image capture was performed using PROVIS AX80 (Olympus, Tokyo, Japan). Samples were classified into high and low CD33^+^ cell infiltration based on the median value as described previously [35].

### Clone selection

The expression vector for the mouse *G-CSF* gene (*pCAmG-CSF*) used in the present study, which was described previously [14], was provided by the RIKEN BRC through the National Bio-Resource Project run by MEXT, Japan. The expression of this gene was driven by the CAG promoter, as reported previously [36, 37]. Transfection was performed using Lipofectamine 2000 (Invitrogen, Carlsbad, CA, USA), according to the manufacturer's instructions. Clonal selection was conducted by adding G418 (Life Technologies, Grand Island, NY, USA) to the medium at a final concentration of 500 μg/ml. ME180 cell lines transfected with the G-CSF-expressing vector (ME180-GCSF) were established.

### Isolation of MDSC

MDSC were isolated from single-cell preparations of ME180-GCSF-bearing Balb/c nu/nu mouse splenocytes using the MDSC isolation kit (mouse) and an MS column (Miltenyi Biotec, Auburn, CA, USA), according to the manufacturer's instructions as described previously [15, 33]. The purity of the isolated cell populations was assessed by flow cytometry, and the frequency of CD11b^+^Gr-1^+^ cells was > 95% [14].

### T-cell proliferation assay

A 24-well plate was coated with 1 μg/well of the anti-CD3e antibody. CD8^+^ T cells were purified from the spleen of a Balb/c mouse using T-cell isolation columns (R&D systems, Minneapolis, MN, USA) according to the manufacturer's instructions and labeled with carboxyfluorescein succinimidyl ester (CFSE). In order to assess the impact of MDSC on CD8^+^ T cell proliferation, CD8^+^ T cells were co-cultured with MDSC with or without E2, ICI 182,780, and JSI-124 for 48 hours. T-cell proliferation was evaluated by flow cytometry. CFSE was purchased from Tonbo Biosciences (San Diego, CA, USA).

### Western blot analysis

Cells were lysed with 1× RIPA Buffer. Lysates (25 μg) were separated by sodium dodecyl sulfate-polyacrylamide gel electrophoresis (SDS-PAGE, Wako Pure Chemical Industries, Ltd., Osaka, Japan) and transferred to polyvinylidene difluoride (PVDF) membranes (GE Healthcare, Little Chalfont, UK). The membranes were incubated with the primary antibodies and then with a corresponding secondary horseradish peroxidase (HRP)-conjugated IgG. Proteins were visualized with an electrochemiluminescent system (PerkinElmer, Waltham, MA, USA).

### Quantitative real-time reverse transcriptase polymerase chain reaction (qRT-PCR)

qRT-PCR was performed using TaqMan probes on a StepOnePlus sequence detection system (Applied Biosystems, Carlsbad, CA, USA). The following TaqMan probes were used: mouse Gapdh (4352339E) and mouse Arginase I (Mm00475988_m1). Relative mRNA expression levels were assessed using the ΔΔCt relative quantification method.

### Chemotaxis assay

Mouse MDSC (5 × 10^5^/well) were seeded in the upper chamber of a 24-well plate. The upper chamber contained a 3-μm pore membrane, which had been pre-coated with fibronectin (Corning, Corning, NY, USA). Tumor-conditioned medium (CM) or 100 ng/ml recombinant mouse CXCL 2 (Wako, Osaka, Japan) in 500 μL of medium were placed in the bottom chamber of the 24-well plate as chemoattractants. In order to obtain CM, supernatants were harvested from confluent cultures of HeLa or MDA-MB-231 cells. After 2 hours’ incubation, the number of cells that had migrated was quantitated by counting the mean number of cells in 4 randomly selected areas per well (magnification: ×200).

### Statistic

PFS was defined as the time from the date of therapy to the date of the first physical or radiographical evidence of disease progression. OS was defined as the time from the date of therapy to the date of death. The survival analysis was based on the Kaplan-Meier method and compared by the Wilcoxon test. Continuous data were compared between groups using the Student's *t*-test or Log-rank test where appropriate. Frequency counts and proportions were compared between the groups using the chi-squared test or a two-tailed Fisher's exact test where appropriate. *P*-values of < 0.05 were considered to be significant. All analyses were performed using the software JMP Pro version 11.0 (SAS Institute, Cary, NC).

### Study approval

Permission to proceed with data acquisition, tumor/blood sample collection, and analyses was obtained from Osaka University Hospital's Institutional Review Board. All procedures involving mice and their care were approved by the Institutional Animal Care and Usage Committee of Osaka University in accordance with institutional and National Institutes of Health guidelines.

## SUPPLEMENTARY MATERIALS FIGURES AND TABLES


