# Supplementary material for: Estrogen stimulates female cancer progression by inducing myeloid-derived suppressive cells: investigations on pregnant and non-pregnant experimental models
Source: Oncotarget. 2019 Mar 8;10(20):1887–902. doi: 10.18632/oncotarget.26711 (PMC6443012; doi:10.18632/oncotarget.26711)
Supplement: Supplementary file 1 [file oncotarget-10-1887-s001.pdf]

Estrogen stimulates female cancer progression by inducing myeloid-derived suppressive cells: investigations on pregnant and non-pregnant experimental models

SUPPLEMENTARY MATERIALS

Supplementary Table 1: Clinicopathological characteristics of cervical cancer patients according to age

		All (*n* = 306)	age ≤ 49 (*n* = 77)	age ≥ 50 (*n* = 229)	*p*-value	
Age, years old	Median (range)	59 (25–86)	42 (25-49)	64 (50-86)		
WBC, /µl	< 10000	263 (85.9%)	58 (22.1%)	205 (77.9%)		
	≥ 10000	43 (14.1%)	19 (44.2%)	24 (55.8%)	0.00219	*
Pelvic node metastasis	Negative	204 (66.7%)	41 (20.1%)	163 (79.9%)		
	Positive	102 (33.3%)	36 (35.3%)	66 (64.7%)	0.0039	*
Histology	SCC	259 (84.6%)	56 (21.6%)	203 (78.4%)		
	Non-SCC	47 (15.4%)	21 (44.7%)	26 (55.3%)	<0.001	*
Tumor size, mm	< 40	58 (19.0%)	12 (20.7%)	46 (79.3%)		
	≥ 40	248 (81.0%)	65 (26.2%)	183 (73.8%)	0.38	

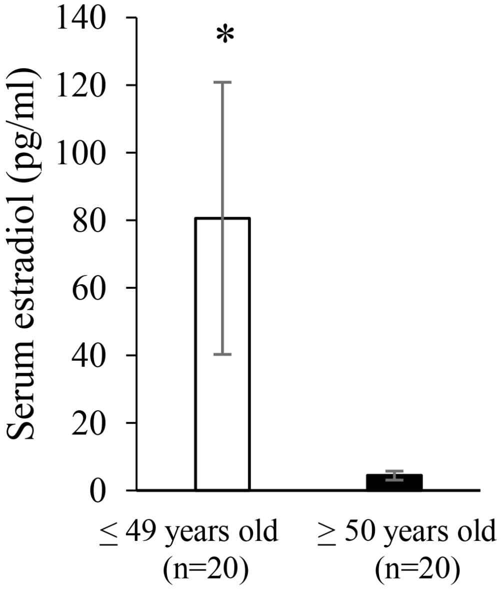

Supplementary Figure 1: Impact of age on serum E2 levels in cervical cancer patients. Serum E2 concentrations in locally-advanced cervical cancer patients (stage IIB-IVA) according to age [≤ 49 years old (n = 20) vs > 50 years old (n = 20)]. Error bars, SE. $^{*}P < 0.05$, Two-sided Student's t-test.

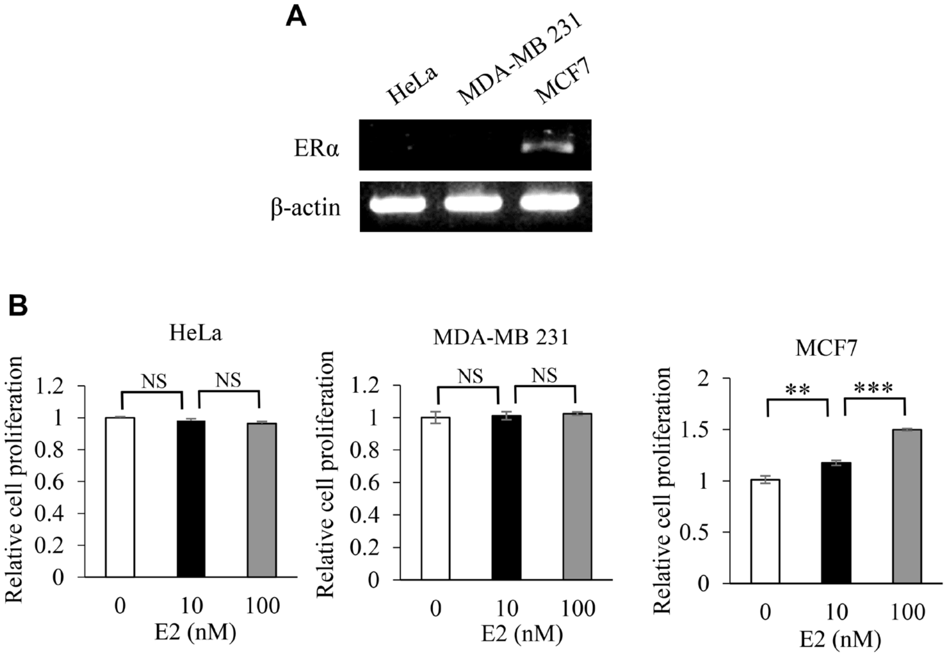

Supplementary Figure 2: Effect of E2 on HeLa, MDA-MB-231, and MCF7 cells. (**A**) The mRNA expression of ERα and β-actin in HeLa, MDA-MB-231, and MCF7 cells assessed by RT-PCR. Each experiment was performed at least three times, and data from one representative experiment are shown. (**B**) *In vitro* effects of E2 on the proliferation of HeLa, MDA-MB-231, and MCF7 cells. Cells were treated with the indicated concentrations of E2 for 48 hours. Cell proliferation was assessed using the MTS assay. Error bars, SE. NS: not significant; $^{**}P < 0.01$, $^{***}P < 0.001$, Two-sided Student's t-test.

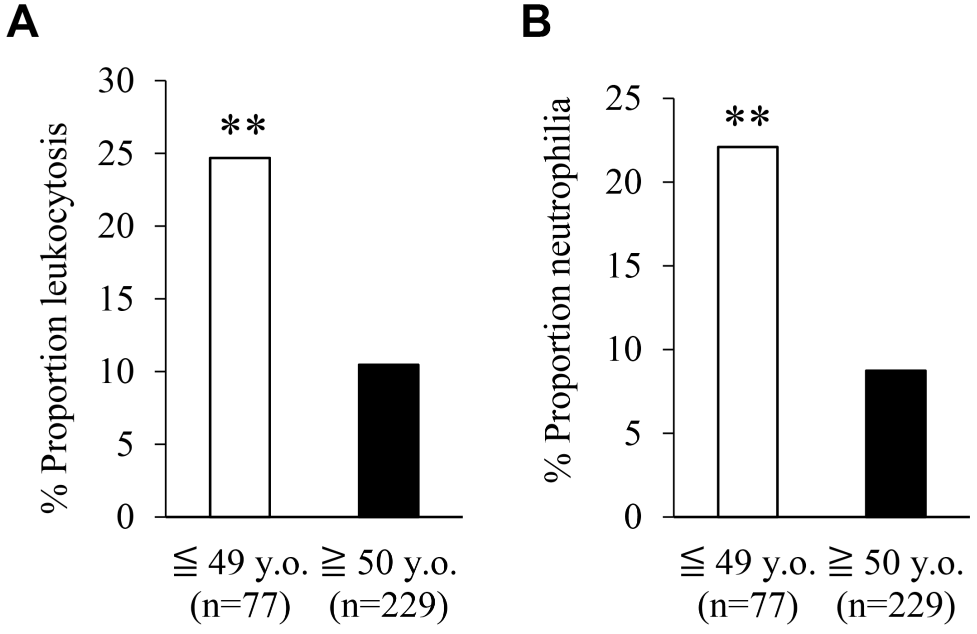

Supplementary Figure 3: Impact of age on blood cell counts in cervical cancer patients. (**A**) The proportion of cervical cancer patients with leukocytosis (> 10000/ml). (**B**) The proportion of cervical cancer patients with neutrophilia (> 8000/ml). $^{**}P < 0.01$, χ^2 test.

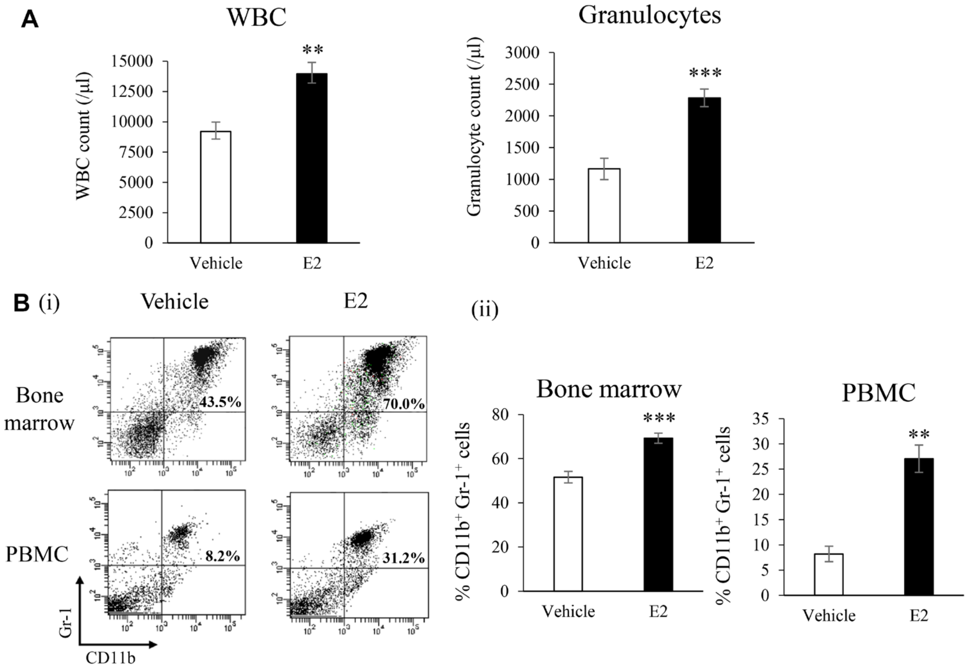

Supplementary Figure 4: Effects of E2 on the induction of MDSC and granulopoiesis *in vivo*. Ovariectomized ICR mice were treated with vehicle (corn oil) or E2 (100 µg/kg/day) once daily for 7 days. BM cells and PBMC were then collected for analyses. (**A**) WBC and granulocyte counts. Error bars, SE. $^{**}P < 0.01$, $^{***}P < 0.001$, Two-sided Student's *t*-test. (**B**) Flow cytometric analysis of the proportion of MDSC (CD11b$^+$Gr-1$^+$). (i) Representative dot plot. The percentages of MDSC were indicated. (ii) Frequencies of MDSC. Error bars, SE. $^{**}P < 0.01$, $^{***}P < 0.001$, Two-sided Student's *t*-test.

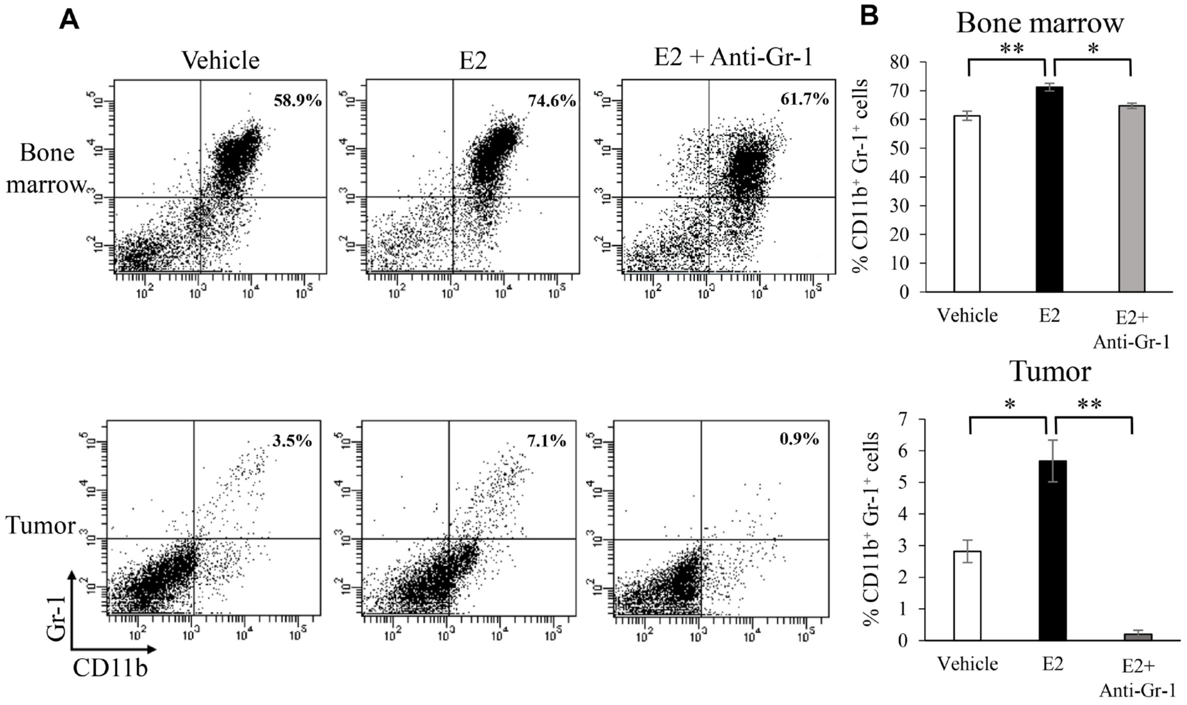

Supplementary Figure 5: Effects of E2 on the induction of MDSC in MDA-MB-231-derived tumor-bearing mice. Ovariectomized Balb/c nu/nu mice were inoculated with MDA-MB-231 and treated with vehicle (0.1% ethanol) or E2 (10 μmol/L) in drinking water from the first day of the inoculation. After tumors reached a volume of approximately 30 mm^3, mice were intraperitoneally treated with the anti-Gr-1-neutralizing antibody (150 μg/mouse) or control IgG (150 μg/mouse) twice a week. At the end of the experiment, mice were killed and the proportions of MDSC ($CD11b^+Gr\text{-}1^+$) in BM and tumor cells were assessed by flow cytometry. (**A**) Representative dot plot. The percentages of MDSC were indicated. (**B**) Frequencies of MDSC. Error bars, SE. $^*P < 0.05$, $^{**}P < 0.01$, Two-sided Student's *t*-test.

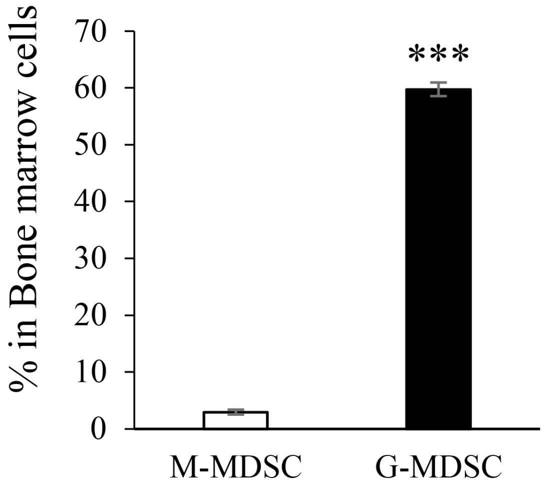

Supplementary Figure 6: MDSC subsets in cancer-bearing mice. Monocytic ($CD11b^+Ly6G^-Ly6C^{high}$) and granulocytic ($CD11b^+Ly6G^+Ly6C^{low}$) MDSC were gated to assess the frequencies of MDSC subsets in the bone marrow of HeLa-derived tumor-bearing mice. Error bars, SE. $^{***}P < 0.001$, Two-sided Student's *t*-test.

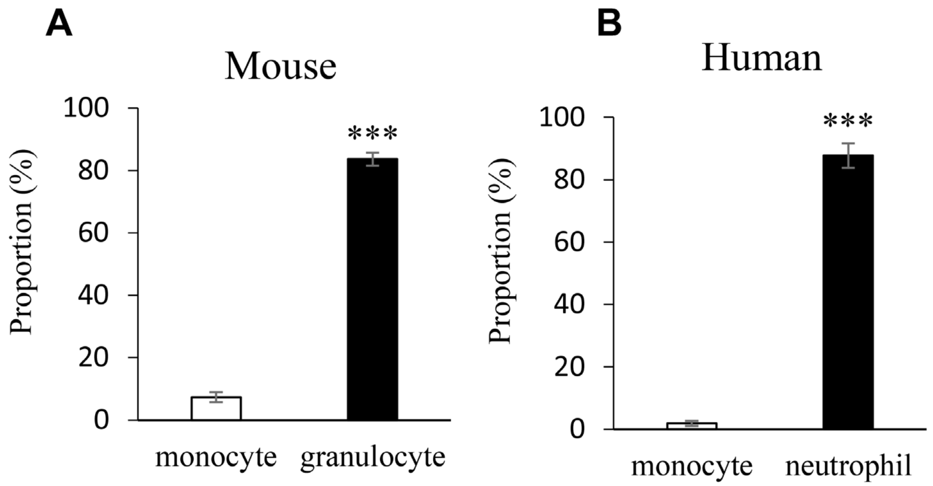

Supplementary Figure 7: MDSC assessed by an automated cell counter. (**A**) Mouse MDSC. Mouse MDSC were assessed by an automated cell counter. Error bars, SE. ***$P < 0.001$, Two-sided Student's *t*-test. (**B**) Human MDSC. MDSC (CD11b+CD33+HLA-DR- cells) obtained from the PBMC of cervical cancer patients were assessed by the automated cell counter. Error bars, SE. ***$P < 0.001$, Two-sided Student's *t*-test.

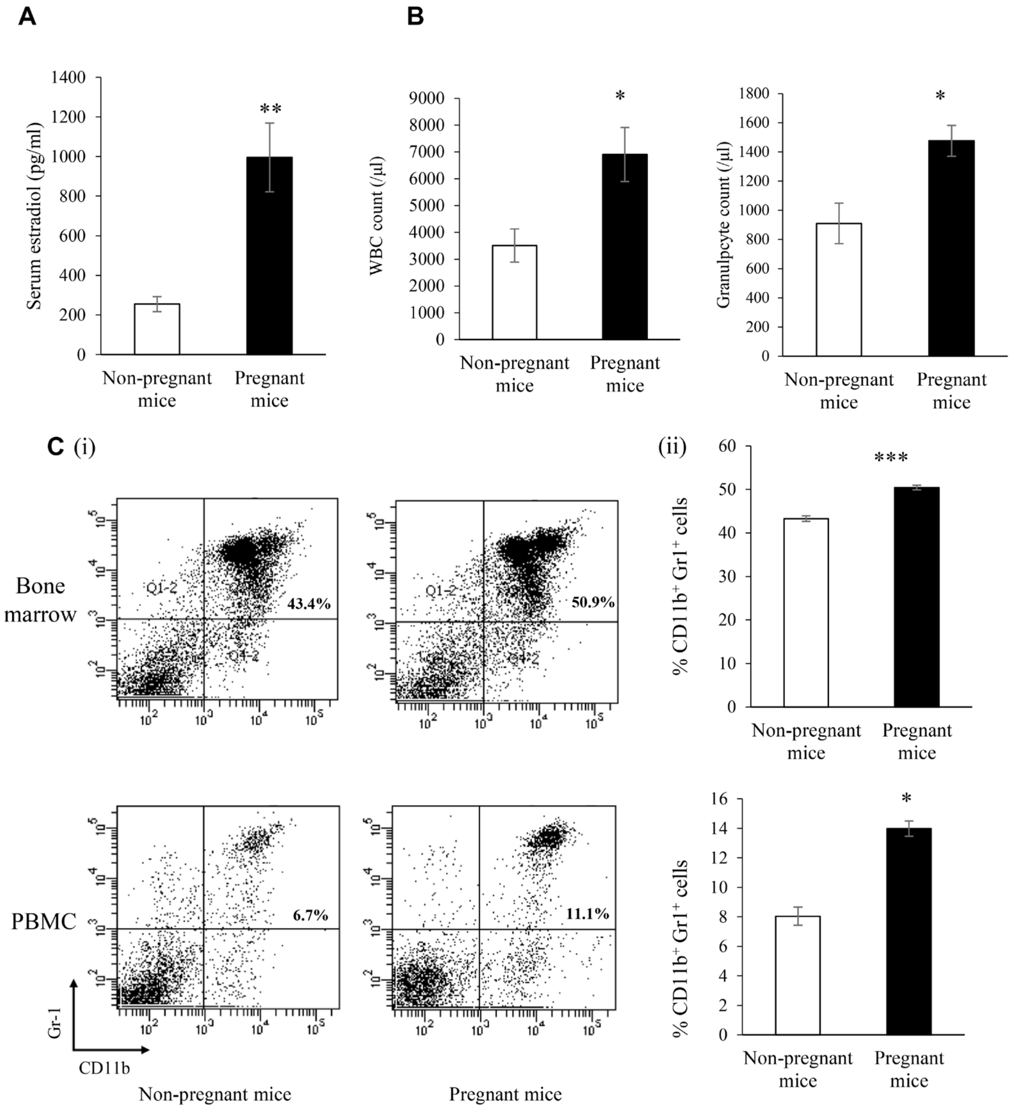

Supplementary Figure 8: Effects of pregnancy on serum E2 levels, granulopoiesis, and the induction of MDSC in non-cancer-bearing mice. BM cells and PBMC were collected from pregnant ICR mice on day 18 of gestation and control ICR mice. The serum concentration of E2 and number of WBC/granulocytes/MDSC were evaluated. (**A**) Serum E2 concentrations measured by ELISA. Error bars, SE. $^{**}P < 0.01$, Two-sided Student's *t*-test. (**B**) WBC/granulocyte counts. Error bars, SE. $^{*}P < 0.05$, $^{**}P < 0.01$, Two-sided Student's *t*-test. (**C**) Proportions of MDSC ($CD11b^{+}Gr\text{-}1^{+}$) in BM and PBMC. (i) Representative dot plot. The percentages of MDSC were indicated. (ii) Frequencies of MDSC. Error bars, SE. $^{*}P < 0.05$, $^{***}P < 0.001$, Two-sided Student's *t*-test.

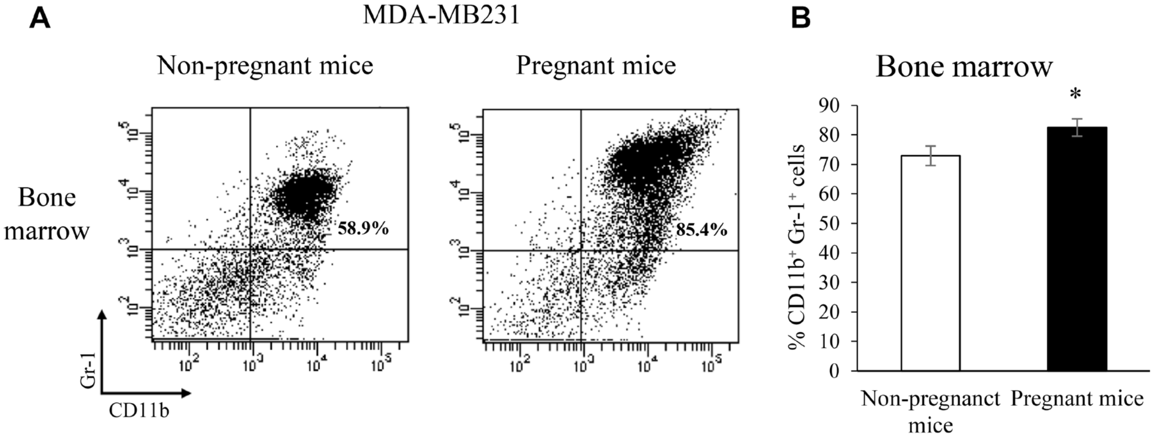

Supplementary Figure 9: Effects of pregnancy on the induction of MDSC in MDA-MB-231-derived tumor-bearing mice. SCID mice were subcutaneously inoculated with MDA-MB-231 cells. When tumors reached a volume of approximately 100 mm^3, half of the mice were impregnated. On day 18 of gestation, mice were sacrificed, BM cells were collected, and the proportions of MDSC ($CD11b^+Gr\text{-}1^+$) were assessed by flow cytometry. (**A**) Representative dot plot. The percentages of MDSC were indicated. (**B**) Frequencies of MDSC. Error bars, SE. $^*P < 0.05$, Two-sided Student's *t*-test.
